# Short-term effect of supplemental yeast extract without or with feed enzymes on growth performance, immune status and gut structure of weaned pigs challenged with *Escherichia coli* lipopolysaccharide

**DOI:** 10.1186/s40104-016-0125-5

**Published:** 2016-11-03

**Authors:** Samuel M. Waititu, Fugui Yin, Rob Patterson, Juan C. Rodriguez-Lecompte, Charles M. Nyachoti

**Affiliations:** 1 Department of Animal Science, University of Manitoba, 201-12 Dafoe Road, Winnipeg, MB R3T 2N2 Canada; 2 Canadian Bio-systems Inc., Calgary, AB T2C 0J7 Canada; 3 Atlantic Veterinary College, University of Prince Edward Island, Charlottetown, PE C1A 4P3 Canada

**Keywords:** Antimicrobial growth promoter, Feed enzymes, Growth performance, Immunity, Nucleotides, Piglets

## Abstract

**Background:**

This study investigated the response of piglets receiving a yeast extract without or with a multi-enzyme mixture compared with an antimicrobial growth promoter (AGP) on performance, immune status and gut structure after an *E. coli* lipopolysaccharide (LPS) challenge. Thirty-six pigs were allotted to six treatments including: a non-challenged control (NCC); LPS-challenged control (CC); CC + AGP; CC + yeast extract; CC + enzymes; and CC + enzymes + yeast extract. On d 7, pigs were bled and thereafter injected with LPS or sterile saline. Blood samples were collected at 6, 48, and 96 h post-challenge. After 96 h post-challenge, pigs were euthanized to obtain duodenal, jejunal and ileal samples.

**Results:**

Overall (d 1 to 11), compared with CC pigs, AGP attenuated the LPS-induced reduction in ADG (*P* = 0.004), ADFI (*P* = 0.03) and gain/feed ratio (*P* = 0.01). At 6 h post-challenge, AGP pigs had lower plasma urea N (PUN; *P* = 0.02) and serum TNF- α concentration (*P* = 0.07), and higher platelet count (*P* = 0.04) and serum IL-10 concentration (*P* = 0.02) than CC pigs. At 48 h post-challenge, AGP pigs had lower PUN (*P* = 0.02) than CC pigs, whereas enzymes + yeast extract interacted non-additively (*P* = 0.001) to reduce PUN. At 96 h post-challenge, AGP pigs had lower PUN (*P* = 0.02) and higher duodenal (*P* = 0.03), jejunal (*P* = 0.01) and ileal (*P* = 0.07) villus height than CC pigs. In addition, enzymes + yeast extract interacted additively and non-additively to reduce ileal IFN-γ (*P* < 0.0001) and IL-10 (*P* = 0.012) expression, respectively. Generally, no differences (*P* > 0.10) were observed between AGP and enzymes + yeast extract pigs on other measured parameters except for the downregulation of ileal IFN-γ (*P* < 0.0001) and TNF-α (*P* = 0.003) in enzymes + yeast extract pigs at 96 h post-challenge.

**Conclusions:**

The LPS challenged piglets receiving enzymes + yeast extract showed beneficial responses in gut structure and immunity commensurate with those receiving antibiotics, though the latter had better overall growth performance.

## Background

Following the ban on sub-therapeutic use of antimicrobial growth promoters (AGP) in animal feed in the European Union [1] and the rising pressure for the same action in USA and Canada [2], the need for potential antibiotic alternatives that can promote piglet growth and health has intensified. Yeast extracts contain various bioactive compounds including nucleotides and cell wall polysaccharides (specifically β-glucan and α-mannan) that are considered beneficial to the growth and gut health of piglets [3], hence yeast extracts have been considered to be a potential in-feed antibiotic alternative. Owing to the presence of various bioactive compounds in yeast extracts, the mechanism by which yeast extracts impact piglet growth and gut health is multifaceted. The main advantage of using yeast extracts as a feed additive is their affordability, a factor which may have less impact on feed cost when compared with the pure sources of its constituent bioactive compounds, thereby facilitating their adoption in the animal feed industry.

Supplementation of yeast extracts rich in nucleotides in piglet starter diets is deemed prudent in replenishing the nucleotide pool sourced from sow milk before weaning [4]. Nucleotides have been suggested to be “conditionally essential” nutrients during early weaning [5] because weaned pigs undergo rapid growth of tissue and organ systems, a process that is heavily dependent on availability of DNA, RNA, and ATP energy, whose synthesis depends on availability of nucleotides. The piglet enterocytes and immune cells have a high rate of replication but this is hampered by their limited capacity for *de novo* synthesis of nucleotides [6], the low concentration of nucleotides in compounded feed [7], and low voluntary feed intake by the piglet due to the transition from liquid to solid feed [8]. This may result in gut atrophy and slow maturation of both the digestive and immune systems, which determines the ability of the piglet to utilize nutrients for growth and to combat pathogens [8].

Supplementation of yeast extracts has been shown to have an immunostimulating effect, which is associated with β-glucans and α-mannans present in yeast cell wall. For instance, it has been reported that yeast β-glucans prevent the elevation in pro-inflammatory cytokines while enhancing the production of anti-inflammatory cytokines when piglets immune system was challenged with lipopolysaccharide (LPS) [9] or *Escherichia coli* [10], whereas supplementing yeast-derived α-mannans in piglet diets has been associated with improved weight gain and feed efficiency, and enhanced function of macrophages in the intestinal lamina propria [11].

It has been suggested that carbohydrases enhance gut health by hydrolyzing complex non-starch polysaccharides (NSP), thereby not only increasing nutrient availability but also inducing a prebiotic effect by releasing fermentable oligosaccharides that are utilized by host gut microbiota as an energy source [12–14]. There is a paucity of studies that simultaneously compare the effects of yeast extracts with AGP or multi-enzyme mixtures supplementation on growth performance and gut health of weaned piglets in response to an inflammatory challenge. Since yeast extracts contain nucleotides and cell wall polysaccharides that are considered to enhance the maturation of the digestive and immune systems of piglets [15], it was hypothesized that given an immune challenge, piglets receiving supplemental yeast extract will have well-balanced immune responses that suppress potentially harmful inflammation compared to challenged control pigs. Consequently, the suppression of harmful inflammation will prevent gut atrophy, encourage voluntary feed intake, and mitigate growth stasis. Moreover, it was hypothesized that a simultaneous supplementation of yeast extract and enzymes will have a synergistic effect of enhancing growth performance of the immune-challenged piglet. Therefore, in the current study, the aim was to determine the response of piglets receiving 0.1 % yeast extract without or with enzymes compared with AGP to an immune system challenge with *E. coli* lipopolysaccharide (LPS) on growth performance, immune status, and gut structure during the immediate post-weaning period.

## Methods

All experimental procedures were reviewed and approved by the University of Manitoba Animal Care Committee, and pigs were handled in accordance with the guidelines described by the Canadian Council on Animal Care [16].

### Animals and experimental design

Thirty-six [Duroc × (Yorkshire × Landrace)] male and female piglets weaned at 21d and with an initial average body weight of 6.89 ± 0.5 kg were used in an 11-d study. Pigs were individually housed in pens in an environmentally controlled nursery building. Each pen was equipped with a feeder, a nipple drinker, and plastic-covered expanded metal floors. The room temperature was maintained at 29 ± 1 °C throughout the study.

On d 1, pigs were randomly assigned to six treatments based on the initial body weight resulting in six replications per treatment. Treatments included: (1) non-challenged control (NCC; pigs fed the basal diet and injected with sterile saline on d 7); (2) LPS-challenged control (CC; pigs fed the basal diet and challenged by injection with *E. coli* LPS on d 7); (3) CC + AGP (pigs fed the basal diet supplemented with an antibiotic growth promoter (AGP) and challenged with *E. coli* LPS on d 7); (4) CC + yeast extract (pigs fed the basal diet supplemented with the yeast extract and challenged with *E. coli* LPS on d 7); (5) CC + enzymes (pigs fed the basal diet supplemented with feed enzymes and challenged with *E. coli* LPS on d 7); and (6) CC + enzymes + yeast extract (pigs fed the basal diet supplemented with enzymes and yeast extract, and challenged with *E. coli* LPS on d 7). The LPS (*E. coli* serotype 055: B5; Sigma Chemical Inc., St Louis, MO, USA) was dissolved in sterile saline and administered intramuscularly at 60 μg/kg body weight, whereas an equivalent amount of sterile saline was administered to NCC pigs. The LPS dosage of 60 μg/kg body weight was based on findings of Rakhshandeh and de Lange [17], which evaluated models of immune system stimulation in pigs and reported that an intramuscular injection of LPS at 60 μg/kg body weight can induce a relatively mild immune system stimulation that can be used to study how nutritional interventions interact with the immune system of pigs.

The basal diet (Table 1) was formulated to meet or exceed the National Research Council [18] requirements for all nutrients. The yeast extract supplement, Maxi-Gen Plus, was supplied by Canadian Bio-systems Inc. (Calgary, AB, Canada) and contained cell wall polysaccharides (21.6 %), CP (32.7 %), carbohydrates (14.3 %) and a mixture of five nucleotides (1.1 %; adenosine monophosphate, cytosine monophosphate, inosine monophosphate, uridine monophosphate and guanosine monophosphate. The AGP supplement, Aureomycin (Pfizer Inc., New York City, NY, USA), supplied 0.10 g chlortetracycline, 0.10 g sulfamethazine and 0.05 g penicillin per kg of feed. The multi-carbohydrase enzymes supplement, Superzyme-OM (Canadian Bio-systems Inc., Calgary, AB, Canada), supplied 2800 U of cellulase, 400 U of mannanase, 50 U of galactanase, 1000 U of xylanase, 600 U of glucanase, 2500 U of amylase and 200 U of protease per kg of diet. All the supplements were top-dressed to the basal diet.

**Table 1:** Composition of the basal diet, as-fed basis

Item	Percent
Ingredient	
Corn	25.25
Wheat	19.00
Soybean meal	20.00
Canola meal	5.00
Fish meal-H	5.00
Dried whey	20.00
Vegetable oil	3.00
Limestone	0.83
Monocalcium phosphate	0.14
Iodized salt	0.26
Vitamin-trace mineral premix^a^	1.00
L-Lys∙HCl	0.35
DL-Methionine	0.10
Threonine	0.07
Calculated nutrient content	
DE, kcal/kg	3573
CP, %	22.3
Ca, %	0.80
Total P, %	0.65
Available P, %	0.43
SID Lys, %	1.36
SID Met, %	0.43
SID Thr, %	0.79
Analyzed composition	
CP, %	23.5
Ca, %	0.86
Total P, %	0.77

^a^Provided the following per kilogram of complete diet: 9000 IU of vitamin A; 1500 IU of vitamin D_3_; 18 mg of vitamin E; 1.5 mg of vitamin K; 250 mg of choline; 30 mg of niacin; 27.5 mg of calcium pantothenate; 9.4 mg of riboflavin; 2 mg of pyridoxine; 25 μg of cyanocobalamin; 80 μg of biotin; 0.5 mg of folic acid; 18 mg of Cu from copper sulfate, 110 mg of Zn from zinc oxide, 0.2 mg of I from calcium iodide, 110 mg of Fe from ferrous sulfate, 50 mg of Mn from manganese dioxide, and 0.3 mg of Se from sodium selenite

From d 1 to 7 and 9 to 11, pigs had free access to feed and water but were pair-fed for 48 h (d 7 to 9) after LPS or sterile saline injection by restricting the feed allowance of all pigs to 4 % of their body weight so as to exclude the possible effects of LPS-induced food intake reduction on gastrointestinal characteristics of the pigs. Feed disappearance and body weight were recorded on d 7 and 11 on pen basis.

### Blood sampling and rectal temperature measurement

Whole blood samples (10 mL per pig) were collected via jugular vein puncture into ethylenediaminetetraacetic acid tubes on d 7 (before LPS challenge), and after 6, 48 and 96 h post LPS or saline injection and were placed on ice until they were processed further. In addition, on d 7 (before LPS challenge) and after 6 h post LPS or saline injection, rectal temperature of each pig was measured and 10-mL blood sample from each pig was collected into a non-heparinized Vacutainer tube (Becton Dickinson, Rutherford, NJ) and incubated at room temperature for 1 h before being centrifuged at 3000 × *g* for 10 min at 4 °C to obtain serum. Serum samples were stored at −80 °C until required for analysis.

### Ileal, duodenal and jejunal tissue sampling

On d 11, that is 96 h post-challenge, all pigs were euthanized. A 5 cm section of duodenum, ileum and jejunum was fixed by immersion in 10 % buffer neutral formalin, whereas 5 g samples of ileum were also collected, stored in RNA later solution (Qiagen, Hilden, Germany), and thereafter stored at −80 °C until required for analysis.

### Sample preparation and chemical analyses

Dietary samples were processed and analyzed for CP, Ca and total P. Crude protein (N × 6.25) was determined according to method 990.03 of AOAC [19] using a combustion analyzer (model CNS-2000; Leco Corp., St. Joseph, MI, USA). Dietary total Ca and P were analyzed following AOAC [19] procedures (method 990.08) using a Varian inductively coupled plasma mass spectrometer (Varian Inc., Palo Alto, CA).

### Histomorphology measurements

The formalin-fixed duodenum, ileum and jejunum tissues were processed at the Veterinary Diagnostic Services Laboratory (Winnipeg, Manitoba, Canada). Villus height and crypt depth were measured at 10× magnification using Axiostar Plus microscope (Carl Zeiss, Oberkochen, Germany) equipped with a camera (Canon Canada Inc., Mississauga, Ontario, Canada) and ImageJ software (National Institutes of Health, Bethesda, MD) in at least 15 well oriented villus and crypt columns. The villus height/crypt depth ratio was calculated.

### Blood cell counts and plasma urea nitrogen (PUN) measurement

Whole blood samples were sent to the Manitoba Veterinary Services Laboratory (Winnipeg, MB, Canada) for complete blood count and PUN analysis. Complete blood count was measured using an automated analyzer (Orthoclinical Johnson & Johnson VITROS 250 Chemistry System; Diamond Diagnostics, Holliston, MA), whereas PUN was measured using a Technicon Autoanalyzer System (Technicon Autoanalyzer Systems, Tarrytown, NY). Both analyses were measured in duplicate.

### Serum TNF-α and IL-10

Serum samples were used to measure the concentration of IL-10 and TNF-α using the quantitative sandwich enzyme-linked immunosorbent assay technique using porcine IL-10 and TNF-α immunoassay kits (R & D Systems, Inc., Minneapolis, MN) according to the manufacturer’s instructions. The optical densities were read on a spectrophotometer (Soft Max Pro 3.1.1; Molecular Devices, Abingdon, Oxfordshire, UK). For each cytokine, the absorbance was measured at 450 nm with the correction wavelength set at 540 nm. Cytokine concentrations were calculated from standards using a 4-parameter logistic curve fit.

### Total RNA extraction and reverse transcription

Total RNA of ileal tissue samples was extracted for the determination of expression profiles of immune response-related cytokines. Briefly, approximately 100 mg of tissue sample was placed in a tube containing 0.5 mL beads and 1 mL TRIZOL reagent (Invitrogen Canada Inc., Burlington, Ontario, Canada) and subjected to bead beating with PowerLyzer (MO BIO Laboratories, Inc., Canada) for 2 min at homogenization level of 2500 psi, and then chilled on ice for 5 min. The above procedure was repeated once. Total RNA was then extracted, washed, and eluted following the manufacturer’s instructions for the TRIZOL reagent. RNA concentration and purity was measured using an ND-1000 spectrophotometer (NanoDrop Technologies, Wilmington, DE, USA). To remove genomic DNA contamination, total RNA was treated with DNase I according to the manufacturer’s protocol. Two micrograms of total RNA with an optical density 260/280 ratio between 1.8 and 2.0 was used for cDNA synthesis using a High Capacity cDNA Reverse transcription Kit (Applied Biosystems, Foster City, CA, USA) with RNase inhibitor according to the manufacturer’s instructions.

### Real-time PCR

Real-time PCR for cDNA templates was carried out using a Bio-Rad CFX Real-time system (BioRad, Hercules, CA, USA). Each reaction mixture was run in duplicate using optical 96-well reaction plates (BioRad, Hercules, CA, USA). Each amplification reaction was carried out with 12.5 μL of iTaq SYBR Green Supermix (BioRad, Hercules, CA, USA) mixed with 1 μL of each primer set of interferon gamma (IFN-γ), tumor necrosis factor-α (TNF-α), interleukin (IL)–1β, IL-10, IL-6, and beta-actin (Table 2), 1 μL of cDNA and 9.5 μL of nuclease-free water. Negative controls were created by replacing cDNA with nuclease-free water. Amplification consisted of initial denaturation for 5 min, followed by 40 cycles of denaturation at 95 °C for 15 s, primer annealing at their individual optimal temperatures (Tables 2 and 3) for 30 s, and an extension step at 72 °C for 30 s. After the amplification, a melting curve analysis with a temperature gradient of 0.1 °C per second from 70 to 95 °C was performed to confirm that only specific products were amplified.

**Table 2:** Primers used for qPCR analysis of immune cytokines

Gene^a^	GenBank reference no.	Amplicon size, bp	T_m_^b^, °C	Primer sequence (5′→3′)
*IFN-γ*	NM_213948.1	231	60	F: GGCCATTCAAAGGAGCATGG R: GCTCTCTGGCCTTGGAACAT
*IL-1β*	NM_214055.1	131	60	F: GCCCATCATCCTTGAAACGTG R: GGAGAGCCTTCAGCATGTGT
*IL-6*	NM_214399.1	149	60	F: TAAGGGAAATGTCGAGGCCG R: TCCACTCGTTCTGTGACTGC
*IL-10*	NM_214041.1	179	60	F: TCCGACTCAACGAAGAAGGC R: AACTCTTCACTGGGCCGAAG
*TNF-α*	NM_214022.1	120	60	F: ATTCAGGGATGTGTGGCCTG R: CCAGATGTCCCAGGTTGCAT
*β-actin*	XM_005670976.1	179	60	F: CGAGGCTCAGAGCAAGAGAG R: GGTTGGCCTTAGGGTTCAGG

^a^ *IFN-γ* interferon gamma, *TNF-α* tumor necrosis factor-α, *IL* interleukin

^b^Annealing temperature

**Table 3:** Effect of supplementing AGP, feed enzymes (ENZ) or yeast extract (YE) without or with ENZ on growth performance of weaned piglets challenged with *E. coli* LPS

		LPS treatments^c^						Factorial^d^			Contrast				
Item^a^	NCC^b^	AGP	CC	YE	ENZ	YE + ENZ	SEM	Main effect YE	Main effect ENZ	YE × ENZ	NCC vs CC	AGP vs CC	AGP vs YE	AGP vs ENZ	AGP vs YE + ENZ
d 1 BW, kg	6.89	6.88	6.90	6.89	6.91	6.86	0.237	NS	NS	NS	NS	NS	NS	NS	NS
d 1 to 7															
ADG, g/d	159	212	150	140	196	164	31.7	NS	NS	NS	NS	NS	NS	NS	NS
ADFI, g/d	191	225	168	160	214	172	24	NS	NS	NS	NS	NS	0.06	NS	NS
G:F, g/g	0.82	0.93	0.77	0.83	0.91	0.87	0.062	NS	NS	NS	NS	NS	NS	NS	NS
d 7 to 11															
ADG, g/d	323	226	130	174	170	160	33.2	NS	NS	NS	0.001	0.07	NS	NS	NS
ADFI, g/d	366	288	214	229	264	249	29.8	NS	NS	NS	0.003	NS	NS	NS	NS
G:F, g/g	0.88	0.8	0.59	0.75	0.64	0.66	0.1	NS	NS	NS	0.07	NS	NS	NS	NS
d 1 to 11															
BW, kg	9.3	9.27	8.07	8.39	8.9	8.78	0.319	NS	0.048	NS	0.02	0.02	0.04	NS	NS
ADG, g/d	241	239	112	162	192	190	27.3	NS	0.017	NS	0.004	0.004	0.03	NS	NS
ADFI, g/d	280	273	177	204	250	210	26.3	NS	NS	NS	0.02	0.03	0.06	NS	0.08
G:F, g/g	0.85	0.88	0.63	0.79	0.77	0.76	0.061	NS	NS	NS	0.02	0.01	NS	NS	NS

^a^ *BW* body weight, *ADG* average daily gain, *ADFI* average daily feed intake, *G:F* gain to feed ratio

^b^ *NCC* non-challenged control pigs that were fed the basal diet and injected with sterile saline on d 7

^c^Pigs fed the basal diet without any additive (*CC* challenged control) or with AGP, YE, ENZ or ENZ + YE and injected with *E. coli* LPS on d 7

^d^ *P*-value for LPS + ENZ × LPS + YE interaction (CC, YE, ENZ and ENZ + YE)

### Statistical analysis

All data were analyzed using the Mixed procedure of SAS (SAS Inst., Inc., Cary, NC). A 2 × 2 factorial analysis of variance was conducted to compare yeast extract and enzymes treatments to determine synergistic effects of yeast extract and enzymes. Preplanned contrasts were used to compare CC with NCC to determine the LPS effect and to compare CC with other treatments, AGP with the yeast extract and AGP with enzymes to determine the effect of the supplements before and after LPS challenge. For all data analyses, significance was defined as *P* < 0.05 and 0.05 < *P* < 0.10 was considered a trend.

## Results

### Growth performance

During d 1 to 7 (pre-challenge), all piglets appeared healthy and no incidence of diarrhea was observed. In addition, there was no difference in ADG and gain/feed ratio (G:F) among treatments except that LPS-challenged AGP pigs tended to have higher ADFI than LPS-challenged pigs receiving the yeast extract (*P =* 0.06; Table 3). Approximately 1 h post-LPS injection, piglets receiving LPS injection irrespective of the dietary treatment began to vomit, became somnolent and developed diarrhea. The piglets recovered from vomiting, somnolence and diarrhea between 24 and 48 h post-LPS injection. During d 7 to 11 (post-challenge), compared with CC pigs, NCC pigs had higher ADG (*P =* 0.001) and ADFI (*P =* 0.003) and tended to have higher G:F (*P =* 0.07), whereas LPS-challenged pigs receiving AGP tended to have higher ADG (*P =* 0.07). Overall (d 1 to 11), pigs receiving enzymes irrespective of the yeast extract had higher body weight (*P* = 0.048) and ADG (*P* = 0.017) than pigs not receiving enzymes. Compared to NCC pigs, LPS-challenge resulted in a 13 % loss in body weight (*P =* 0.02), 56 % reduction in ADG (*P =* 0.004), a 37 % reduction in ADFI (*P =* 0.02) and a 26 % reduction in G:F (*P =* 0.02) of CC pigs. Compared to CC pigs, supplementation of AGP improved body weight (*P =* 0.02), ADG (*P =* 0.004), ADFI (*P =* 0.03) and G:F (*P =* 0.01). In addition, LPS-challenged pigs receiving AGP had higher body weight (*P =* 0.04) and ADG (*P =* 0.03) than LPS-challenged pigs receiving the yeast extract and tended to have higher ADFI than LPS-challenged pigs receiving the yeast extract (*P =* 0.06) or enzymes + yeast extract (*P =* 0.08). There were no differences between AGP and enzymes + yeast extract pigs on ADG and G:F.

### Plasma urea N content and blood cells count

No effect of dietary treatment was observed for PUN pre-challenge (Table 4). After 6 h post-challenge, pigs receiving yeast extract irrespective of enzymes had lower PUN (*P* = 0.03) than pigs not receiving yeast extract, CC pigs had higher PUN than NCC pigs (*P =* 0.01) and LPS-challenged pigs receiving AGP (*P =* 0.02). After 48 h post-challenge, pigs receiving yeast extract irrespective of enzymes had lower PUN (*P* = 0.01) than pigs not receiving yeast extract, CC pigs tended to have higher PUN than NCC pigs (*P =* 0.08) and LPS-challenged pigs receiving AGP (*P =* 0.02). In addition, LPS-challenged pigs receiving the yeast extract tended to have lower PUN than those receiving AGP (*P =* 0.07). Moreover, an enzymes × yeast extract interaction (*P* = 0.001) was observed showing a non-additive effect of enzymes and the yeast extract in reducing the PUN of pigs. After 96 h post-challenge, CC pigs had higher PUN than NCC (*P =* 0.01) and LPS-challenged pigs receiving AGP (*P =* 0.02), whereas LPS-challenged pigs receiving enzymes + yeast extract tended to have higher PUN than those receiving AGP (*P =* 0.09). In addition, a tendency towards an enzymes × yeast extract interaction (*P* = 0.057) was observed showing a non-additive effect of enzymes and the yeast extract in reducing the PUN of pigs.

**Table 4:** Effect of supplementing AGP, feed enzymes (ENZ) or yeast extract (YE) without or with ENZ on PUN (mmol/L) and count of white blood cells (WBC, 10^9^/L), red blood cells (RBC, 10^12^/L) and platelets (10^9^/L) of weaned piglets challenged with *E. coli* LPS

		LPS treatments^b^						Factorial^c^			Contrast				
Item	NCC^a^	AGP	CC	YE	ENZ	YE + ENZ	SEM	Main effect YE	Main effect ENZ	YE × ENZ	NCC vs CC	AGP vs CC	AGP vs YE	AGP vs ENZ	AGP vs YE + ENZ
PUN															
Pre-challenge	2.34	2.28	2.95	2.08	2.52	2.57	0.412	NS	NS	NS	NS	NS	NS	NS	NS
6 h post-challenge	2.12	2.58	4.18	2.5	3.42	2.6	0.478	0.03	NS	NS	0.01	0.02	NS	NS	NS
48 h post-challenge	2.16	1.93	2.97	1.24	1.6	1.83	0.285	0.01	NS	0.001	0.08	0.02	0.07	NS	NS
96 h post-challenge	1.66	1.78	3.2	2.1	2.55	2.77	0.384	NS	NS	0.057	0.01	0.02	NS	NS	0.09
WBC															
Pre-challenge	16.9	18	17.8	15	14.1	19.3	2.21	NS	NS	0.062	NS	NS	NS	NS	NS
6 h post-challenge	19.7	10.2	6.5	12.2	7.6	15.5	1.98	0.003	NS	NS	0.0004	NS	NS	0.07	0.07
48 h post-challenge	18.2	16.8	17.3	17.5	16.2	19.7	1.68	NS	NS	NS	NS	NS	NS	NS	NS
96 h post-challenge	19.6	16.7	16.9	16.3	14.5	18.2	1.3	NS	NS	NS	NS	NS	NS	NS	NS
RBC															
Pre-challenge	5.96	5.54	6.04	6.09	6.04	6.06	0.228	NS	NS	NS	NS	NS	NS	NS	NS
6 h post-challenge	5.91	5.68	6.01	6.62	6.33	5.97	0.255	NS	NS	0.090	NS	NS	0.01	NS	NS
48 h post-challenge	5.59	4.96	5.39	5.41	5.48	5.25	0.184	NS	NS	NS	NS	NS	0.07	NS	NS
96 h post-challenge	5.95	5.18	5.32	5.6	5.46	5.22	0.19	NS	NS	NS	0.02	NS	NS	NS	NS
Platelet															
Pre-challenge	429	534	450	483	447	415	44.8	NS	NS	NS	NS	NS	NS	NS	NS
6 h post-challenge	413	309	196	350	205	278	37.3	0.001	NS	NS	0.0002	0.04	NS	NS	NS
48 h post-challenge	445	345	350	338	322	345	29.9	NS	NS	NS	0.05	NS	NS	NS	NS
96 h post-challenge	449	446	386	520	427	507	61.3	0.06	NS	NS	NS	NS	NS	NS	NS

^a^ *NCC* non-challenged control pigs that were fed the basal diet and injected with sterile saline on d 7

^b^Pigs fed the basal diet without any additive (*CC* challenged control) or with AGP, YE, ENZ or ENZ + YE and injected with *E. coli* LPS on d 7

^c^ *P*-value for LPS + ENZ × LPS + YE interaction (CC, YE, ENZ and ENZ + YE)

A tendency towards an enzymes × yeast extract interaction (*P* = 0.062) was observed pre-challenge showing a non-additive effect of enzymes and the yeast extract in increasing the white blood cell count of pigs. After 6 h post-challenge, pigs receiving yeast extract irrespective of enzymes had higher white blood cell count (*P* = 0.003) than pigs not receiving yeast extract, CC pigs had lower white blood cell count than NCC (*P =* 0.0004). The LPS-challenged pigs receiving AGP tended to have higher white blood cell count than those receiving enzymes (*P =* 0.07) and lower white blood cell count than those receiving enzymes + yeast extract (*P =* 0.07). After 48 and 96 h post-challenge, treatments had no effect on white blood cell count.

No effect of dietary treatment was observed for red blood cells count before LPS challenge. After 6 h post-challenge, LPS-challenged pigs receiving AGP had lower red blood cell count (*P =* 0.01) than challenged pigs receiving the yeast extract and a tendency towards an enzymes × yeast extract interaction (*P* = 0.090) was observed showing a non-additive effect of enzymes and the yeast extract in decreasing the red blood cell count of pigs. After 48 h post-challenge, LPS-challenged pigs receiving AGP tended to have higher (*P =* 0.07) red blood cell count than challenged pigs receiving the yeast extract, whereas after 96 h post-challenge, compared to NCC pigs, red blood cell count was lower in CC pigs (*P =* 0.02).

No effect of dietary treatment was observed for platelet count pre-challenge. After 6 h post-challenge, pigs receiving yeast extract irrespective of enzymes had higher platelet count (*P* = 0.001) than pigs not receiving yeast extract. The platelet count of CC pigs was lower than of NCC pigs (*P =* 0.0002) and LPS-challenged pigs receiving AGP. After 48 h post-challenge, pigs receiving yeast extract irrespective of enzymes tended to have a higher platelet count (*P* = 0.06) than pigs not receiving yeast extract. The platelet count of CC pigs was lower (*P* = 0.05) than of NCC pigs and was not influenced by treatments after 96 h post-challenge.

### Duodenum, jejunum and ileum histomorphology

After 96 h post-challenge, pigs receiving yeast extract irrespective of enzymes tended to have a higher duodenal villus height (*P* = 0.098) than pigs not receiving yeast extract. Pigs receiving enzymes irrespective of the yeast extract had higher duodenal villus height (*P* = 0.004) than pigs not receiving enzymes, whereas the duodenal villus height for CC pigs was lower than that of NCC (*P =* 0.002) and LPS-challenged pigs receiving AGP (*P =* 0.03; Table 5). The duodenal crypt depth for CC pigs was lower than that of NCC pigs (*P =* 0.02). Pigs receiving yeast extract irrespective of enzymes had higher jejunal villus height (*P* = 0.049) and jejunal VH:CD (*P* = 0.03) than pigs not receiving yeast extract, whereas pigs receiving enzymes irrespective of the yeast extract had higher jejunal villus height (*P* = 0.047) and tended to have higher jejunal VH:CD (*P* = 0.07) than pigs not receiving enzymes. In addition, CC pigs had shorter jejunal villus height than NCC pigs (*P* < 0.0001) and LPS-challenged pigs receiving AGP (*P =* 0.01). Moreover, compared with CC pigs, the jejunal VH:CD ratio was higher in NCC (*P =* 0.0003) pigs and LPS-challenged pigs receiving AGP (*P =* 0.01). The ileal villus height of CC pigs was shorter than of NCC pigs (*P =* 0.04) and tended to be shorter than that of LPS challenged pigs receiving AGP (*P =* 0.07).

**Table 5:** Effect of supplementing AGP, feed enzymes (ENZ) or yeast extract (YE) without or with ENZ on ileum, jejunum and duodenum histomorphology of weaned piglets after 96 h of challenge with *E. coli* LPS

		LPS treatments^c^						Factorial^d^			Contrast				
Item^a^	NCC^b^	AGP	CC	YE	ENZ	YE + ENZ	SEM	Main effect YE	Main effect ENZ	YE × ENZ	NCC vs CC	AGP vs CC	AGP vs YE	AGP vs ENZ	AGP vs YE + ENZ
Duodenum, μm															
Villus height	532	493	410	480	520	538	24.7	0.098	0.004	NS	0.002	0.03	NS	NS	NS
Crypt depth	322	308	277	305	314	316	13.1	NS	NS	NS	0.02	NS	NS	NS	NS
VH:CD	1.65	1.61	1.50	1.58	1.68	1.70	0.088	NS	NS	NS	NS	NS	NS	NS	NS
Jejunum, μm															
Villus height	465	400	293	385	385	412	26.3	0.049	0.047	NS	<.0001	0.01	NS	NS	NS
Crypt depth	277	269	255	275	282	273	13.2	NS	NS	NS	NS	NS	NS	NS	NS
VH:CD	1.70	1.50	1.15	1.40	1.37	1.51	0.092	0.03	0.07	NS	0.0003	0.01	NS	NS	NS
Ileum, μm															
Villus height	329	323	272	294	308	306	18.8	NS	NS	NS	0.04	0.07	NS	NS	NS
Crypt depth	265	259	244	233	268	249	13.6	NS	NS	NS	NS	NS	NS	NS	NS
VH:CD	1.24	1.26	1.11	1.26	1.17	1.23	0.067	NS	NS	NS	NS	NS	NS	NS	NS

^a^ *VH:CD* villus height/crypt depth; means are averages of at least 15 well oriented villus and crypt columns

^b^ *NCC* non-challenged control pigs that were fed the basal diet and injected with sterile saline on d 7

^c^Pigs fed the basal diet without any additive (*CC* challenged control) or with AGP, YE, ENZ or ENZ + YE and injected with *E. coli* LPS on d 7

^d^ *P*-value for LPS + ENZ × LPS + YE interaction (CC, YE, ENZ and ENZ + YE)

### Rectal temperature, serum and ileum cytokines

Before LPS challenge, pigs receiving enzymes irrespective of the yeast extract tended to have higher rectal temperature (*P* = 0.06) than pigs not receiving enzymes, whereas no effect of treatments was observed for concentration of serum TNF-α and IL-10 (Table 6). After 6 h post-challenge, rectal temperature was higher in CC pigs (*P =* 0.003) compared with NCC pigs. In addition, pigs receiving yeast extract irrespective of enzymes had a lower concentration of serum TNF-α (*P* = 0.001) and higher concentration of serum IL-10 (*P* = 0.004) than pigs not receiving yeast extract. Compared with CC pigs, the concentration of serum TNF-α was lower in NCC pigs (*P* < 0.0001) and LPS-challenged pigs receiving AGP (*P* = 0.07), whereas the serum IL-10 concentration was lower in NCC pigs (*P* = 0.001) but higher in LPS-challenged pigs receiving AGP (*P* = 0.02).

**Table 6:** Effect of supplementing AGP, feed enzymes (ENZ) and yeast extract (YE) without or with ENZ on the rectal temperature (°C), concentration of serum (pg/mL) cytokines and fold changes (log_2_) of ileal immune cytokines of weaned piglets challenged with *E. coli* LPS

		LPS treatments^b^						Factorial^c^			Contrast				
Item	NCC^a^	AGP	CC	YE	ENZ	YE + ENZ	SEM	Main effect YE	Main effect ENZ	YE × ENZ	NCC vs CC	AGP vs CC	AGP vs YE	AGP vs ENZ	AGP vs YE + ENZ
Rectal temperature															
Pre-challenge	38.9	39.2	39.1	39.0	39.2	39.2	0.13	NS	0.06	NS	NS	NS	NS	NS	NS
6 h post-challenge	39.2	39.9	39.9	39.8	40.1	40.1	0.15	NS	NS	NS	0.003	NS	NS	NS	NS
Serum TNF-α															
Pre-challenge	98	88	96	77	102	93	9.3	NS	NS	NS	NS	NS	NS	NS	NS
6 h post-challenge	111	1032	1369	854	1621	933	120.8	0.001	NS	NS	<.0001	0.07	0.85	0.003	NS
Serum IL-10															
Pre-challenge	18	16	29	22	36	27	15.1	NS	NS	NS	NS	NS	NS	NS	NS
6 h post-challenge	16	157	99	153	86	156	15.3	0.004	NS	NS	0.001	0.02	NS	NS	NS
Ileum cytokines, 96 h post-challenge															
IFN-γ	1.00	1.54	1.46	1.51	2.15	0.54	0.152	<.0001	NS	<.0001	0.04	NS	NS	0.008	<.0001
TNF-α	1.00	2.52	2.40	1.37	1.60	1.41	0.244	0.013	0.099	0.073	0.0003	NS	0.002	0.013	0.003
IL-1β	1.00	0.95	1.63	1.02	0.77	0.58	0.154	0.025	0.001	NS	0.01	0.004	NS	NS	NS
IL-6	1.00	0.86	0.85	0.83	0.69	1.12	0.130	NS	NS	NS	NS	NS	NS	NS	NS
IL-10	1.00	0.60	1.31	0.74	0.71	0.76	0.123	0.033	0.019	0.012	0.09	0.0003	NS	NS	NS

^a^ *NCC* non-challenged control pigs that were fed the basal diet and injected with sterile saline on d 7

^b^Pigs fed the basal diet without any additive (*CC* challenged control) or with AGP, YE, ENZ or ENZ + YE and injected with *E. coli* LPS on d 7

^c^ *P*-value for LPS + ENZ × LPS + YE interaction (CC, YE, ENZ and ENZ + YE)

After 96 h post-challenge, pigs receiving yeast extract irrespective of enzymes had a lower expression of IFN-γ (*P* > 0.0001), TNF-α (*P* = 0.013), IL-1β (*P* = 0.025) and IL-10 (*P* = 0.033) than pigs not receiving yeast extract. On the other hand, pigs receiving enzymes irrespective of the yeast extract had a lower expression of IL-1β (*P* = 0.001) and IL-10 (*P* = 0.019), and a tendency towards lower expression of TNF-α (*P* = 0.099) than pigs not receiving enzymes. Compared to NCC pigs, the CC pigs had higher expression of ileal IFN-γ (*P =* 0.04). An enzymes × yeast extract interaction (*P* < 0.0001) was observed showing an additive effect of enzymes and the yeast extract in downregulating the expression of ileal IFN-γ in LPS-challenged pigs. Compared with pigs receiving AGP, the ileal IFN-γ expression was higher (*P* = 0.008) in LPS-challenged pigs receiving enzymes but lower (*P* < 0.0001) in pigs receiving enzymes + yeast extract.

After 96 h post-challenge, the CC pigs had a higher expression of ileal TNF-α (*P =* 0.0003) than NCC pigs. A tendency towards an enzymes × yeast extract interaction (*P* = 0.073) was observed showing a non-additive effect of enzymes and the yeast extract in downregulating the expression of ileal TNF-α in LPS-challenged pigs. Compared with LPS challenged pigs receiving AGP, TNF-α expression was lower in LPS-challenged pigs receiving yeast extract (*P* = 0.002), enzymes (*P* = 0.013) or enzymes + yeast extract (*P* = 0.003).

After 96 h post-challenge, compared with CC pigs, the expression of ileal IL-1β was lower in NCC pigs (*P =* 0.01) and in LPS-challenged pigs receiving AGP (*P* = 0.004). The CC pigs tended to have a higher expression of ileal IL-10 (*P =* 0.09) than NCC pigs. An enzymes × yeast extract interaction (*P* = 0.012) was observed showing a non-additive effect of enzymes and the yeast extract in downregulating the expression of ileal IL-10 in LPS-challenged pigs. Compared with CC pigs, IL-10 expression was lower in LPS-challenged pigs receiving AGP (*P* = 0.0003).

## Discussion

The AGP, yeast extract and enzymes supplements did not affect performance during the first 7 d of weaning. Sauer et al. [20] and Andrés-Elias et al. [21] supplemented 0.1 and 0.15 % yeast extract in piglet starter diets, respectively, and reported no effect of yeast extract on growth performance. Between d 7 and 11 (post-challenge), the LPS challenge reduced ADG, ADFI and G:F of CC pigs. This observation is consistent with the findings of Liu et al. [22] and Liu et al. [23] showing that LPS challenge negatively impacts growth performance in pigs. Although none of the supplements was able to fully protect the pigs from weight loss or to increase their feed intake between d 7 and 11, the LPS impact on ADG tended to be reduced in AGP receiving pigs compared with other LPS-challenged treatments. This beneficial effect of AGP on growth performance was further seen in the overall experimental period (d 1 to 11) with AGP pigs having better ADG, ADFI and GF than CC pigs, and tended to have better G:F than pigs supplemented with yeast extract. These observations suggest that AGP supplemented pigs had better recovery of weight and feed intake after LPS challenge than those supplemented with yeast extract. Weber et al. [24] and Skinner et al. [25] reported that AGP improves ADG during the nursery phase, whereas Wu et al. [26] reported that piglets receiving in-feed AGP and challenged with enterotoxigenic *E. coli* had higher ADG and G:F than control. Niewold [27] suggested that AGP improve gut health and growth performance of livestock by reducing the gut pathogen load and competition for nutrients between the host and gut microbes. This effect may also be associated with the immunoregulatory role of AGP [28]. Although enzymes supplementation tended to improve ADG (d 1 to 11), there was no synergistic effect between yeast extract and enzymes on ADG.

The reduced white blood cell and platelet counts in CC pigs is due to LPS induced autoimmunity and suggests an increased incidence or severity of infection [29, 30]. Platelets play a crucial role in blood clotting and as the first responders to sites of vascular injury or pathogen invasion, platelets play multiple roles in the modulation of both innate and adaptive immunity thereby boosting host defense against infection [30]. The observation that LPS-challenged pigs receiving yeast extract maintained higher white blood cell and platelet count than CC pigs and higher red blood cell count than LPS-challenged pigs receiving AGP at 6 h post-challenge suggests that yeast extract supplementation either had a role in preventing blood cells death or sustained moderate blood cell formation during the challenge period.

A 100 μg/kg dosage of *E. coli* LPS has been shown to induce gut injury in pigs [23, 31]. In the present study, gut injury was observed in piglets that received 60 μg/kg of *E. coli* LPS. Supplementing yeast extract to pigs without or with enzymes reduced villus atrophy in the jejunum and duodenum, and the villus height/crypt depth ratio in the jejunum when compared with the non-challenged control pigs, and the effect was similar to that of AGP supplementation. The increase in villus height and villus/crypt ratio indicates an improvement in the digestion and absorption of nutrients [31, 32]. Enterocytes have limited capacity for *de novo* nucleotides synthesis [33, 34] and thus the increased pool of dietary nucleotides by yeast extract supplementation promoted intestinal tissue growth and development. In addition, pathogens with mannose-specific type-1 fimbriae, such as *E. coli* and *Salmonella*, bind with the α-mannans instead of attaching to intestinal epithelial cells, which may reduce inflammation that leads to intestinal villi atrophy [35].

Injection of LPS induces fever in pigs by stimulating the production of cytokines such as IL-1, TNF-α and IFN-γ, which are regarded as endogenous mediators of fever [36]. In this study, 6 h post-challenge CC pigs showed a significant rise in body temperature compared to NCC pigs as also observed by Pi et al. [37] and none of the supplements protected the pigs from fever development. The concentration of serum TNF-α and IL-10 were measured as indicators of systemic pro- and anti-inflammation responses, respectively. Pigs receiving AGP or yeast extract without or with enzymes had less concentration of serum TNF-α and higher concentration of IL-10 than CC pigs, hence, implying that AGP and enzymes + yeast extract supplementation lowered pro-inflammation of the immune system. The ileum immune response was also influenced by both LPS and the supplements. The observed upregulation of ileal TNF-α and IL-1β 96 h post-challenge in the CC pigs compared to NCC pigs suggests development of inflammation in CC pigs, which if prolonged leads to tissue damage such as the observed villus atrophy in these pigs [38]. In contrast, pigs in the yeast extract supplemented treatments had downregulated levels of expression of these pro-inflammatory cytokines thus suggesting that the yeast extract induced beneficial immunoregulatory responses post-challenge that was evidenced by lower expression of TNF-α and IL-1β, and consequently that of IL-10 compared to the LPS-challenged control group. The overproduction of proinflammatory cytokines is associated with anorexia development, and may explain the observed reduction in ADFI in LPS-challenged pigs in this study [39, 40].

In the present study, LPS-challenged control pigs had higher PUN than non-challenged control pigs. This is consistent with the study of Webel et al. [41] reporting that LPS challenge increases the PUN level in piglets due to muscle proteolysis as a result of increased inflammation. Owusu-Asiedu et al. [42] suggested that the released amino acids due to inflammation may be channeled to the liver to synthesize acute phase proteins and/or to serve as an energy source. Supplementing yeast extract without or with enzymes had similar effects as AGP in reducing PUN level compared with the LPS-challenged control. The reduction in PUN level is an indicator of reduced muscle protein breakdown [41] and is consistent with the observed reduced production of proinflammatory cytokines in the yeast extract and AGP receiving pigs. These observations suggest that supplementing yeast extract or AGP was beneficial to piglets and reduced the severity of the LPS challenge. Such a response may promote efficiency in utilization of dietary amino acids for proteinaceous tissue growth [39].

Taken together, after the immune system stimulation using *E. coli* LPS, the growth performance, platelets count, villus height of the jejunum and duodenum, and immune status of LPS-challenged control pigs was negatively affected compared with the non-challenged control. Compared to LPS-challenged control, supplementing yeast extract + enzymes lowered and increased the expression of ileal IFN-γ and IL-10, respectively, and improved histomorphological measurements but did not affect growth performance. Most of the observed enzymes × yeast extract interactions or tendencies towards interaction were non-additive except that of lowering ileal IFN-γ. The most pronounced beneficial effects of yeast extract supplementation were on lowering PUN, villus atrophy, and levels of pro-inflammatory cytokines, and maintaining blood cell counts close to that of non-challenged control pigs.

The immunomodulating effect of the yeast extract is multifaceted and may be associated with the nucleotides and cell wall polysaccharides present in the yeast extract. In response to the inflammatory challenge, the nucleotides sourced from the yeast extract can be channeled to influence the development and maturation of important tissues of the immune system and the intestinal mucosa. This supports the suggestion by Gaskins et al. [43] that the weaned pig first invests substantially in defensive efforts to sequester gut microbes away from the epithelial surface, and second to quickly mount immune responses against microbes that breach epithelial defenses. This re-direction of nutrients from growth towards fortification of the immune system and maturation of gut structures may explain why beneficial responses on growth performance may not be observed within a short term of supplementing the yeast extract nucleotides. However, a faster maturation of the gut and immune system tissues, may in the long-run support faster growth of the pig, hence the effect of nucleotides may be long-lasting. On the other hand, there is evidence that yeast β-glucans are associated with downregulation and upregulation of the expression of pro-inflammatory and anti-inflammatory cytokines, respectively, when piglets immune system is challenged with LPS [9] or *E. coli* [10], and that yeast-derived α-mannans has been associated with improved enhanced function of macrophages in the intestinal lamina propria [11]. The yeast cell wall polysaccharides may also provide binding sites for pathogens hence mediating their elimination from the gut [44].

## Conclusions

In conclusion, LPS-challenged piglets fed diets supplemented with yeast extract without or with enzymes expressed similar beneficial responses as those fed diets with AGP in terms of lowering PUN concentration, reducing duodenal and ileal villi atrophy, and downregulating serum and ileal proinflammatory cytokines. This suggests that supplementation of the yeast extract was associated with promotion of the health of piglets during the immediate post-weaning period.

## Abbreviations

ADFI: Average daily feed intake
ADG: Average daily gain
AGP: Antimicrobial growth promoters
CP: Crude protein
G:F: Gain/feed ratio
IFN-γ: Interferon gamma
IL: Interleukin
LPS: *E. coli* lipopolysaccharide
PUN: Plasma urea nitrogen
TNF-α: Tumor necrosis factor alpha
